# Ultrasensitive magnetic resonance imaging of systemic reactive oxygen species *in vivo* for early diagnosis of sepsis using activatable nanoprobes†
†Electronic supplementary information (ESI) available: Experimental methods and supporting figures. See DOI: 10.1039/c8sc04961k


**DOI:** 10.1039/c8sc04961k

**Published:** 2019-02-21

**Authors:** Huan Wang, Dongqin Yu, Bo Li, Zhen Liu, Jinsong Ren, Xiaogang Qu

**Affiliations:** a State Key Laboratory of Rare Earth Resources Utilization , Laboratory of Chemical Biology , Changchun Institute of Applied Chemistry , Chinese Academy of Sciences , Changchun , 130022 , P. R. China . Email: jren@ciac.ac.cn ; Email: xqu@ciac.ac.cn; b University of Science and Technology of China , Hefei , 230029 , P. R. China; c Department of Radiology , The Second Hospital of Jilin University , Changchun , Jilin 130041 , P. R. China; d Beijing Advanced Innovation Center for Soft Matter Science and Engineering , Beijing University of Chemical Technology , Beijing , 100029 , P. R. China . Email: liuganxuan@mail.buct.edu.cn

## Abstract

Current diagnostic methods for sepsis lack required speed or precision, often failing to make timely accurate diagnosis for early medical treatment. The systemic excess generation of reactive oxygen species (ROS) during sepsis has been considered as an early indicator of sepsis. Herein, we present the rational design of novel activatable nanoprobes (ROS CAs) composed of a clinically approved iron oxide core, Gd–DTPA, and hyaluronic acid (HA) that can image ROS down to sub-micromolar concentrations *via* magnetic resonance imaging (MRI), and use them as sensitive contrast agents for sepsis evaluation. Such a well-defined nanostructure allows them to undergo ROS-triggered degradation and release Gd–DTPA in the presence of ROS, leading to the recovery of the quenched *T*_1_-weighted MRI signal with fast response. With outstanding sensitivity and unlimited tissue penetration depth, ROS CAs are capable of imaging systemic ROS overproduction in mice with early sepsis. Moreover, by using these well-prepared ROS CAs, the severity of the sepsis can be rapidly evaluated by monitoring the systemic ROS levels *in vivo*. Overall, the present study will not only provide a new strategy to aid in the early diagnosis and risk assessment of sepsis, but also offer valuable insight for the study of sepsis and ROS biology.

## Abstract 2

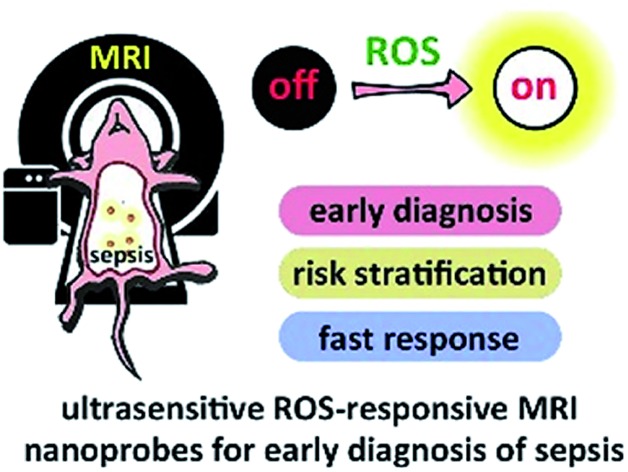
Novel ROS-activated contrast agents are designed for magnetic resonance imaging of ROS for early diagnosis of sepsis.

## Introduction

Sepsis is a type of systemic inflammatory response syndrome induced by infections, and is becoming a major cause of admission to the intensive care unit with a high in-hospital mortality rate. If medical treatment is delayed, sepsis will rapidly develop into septic shock, multiple organ dysfunction syndrome (MODS), and death.^1^,^2^ Owing to the emergence of drug-resistant pathogens and wide use of immunosuppressants, the incidence of sepsis continues to increase.^3^,^4^ Because of the acute nature of sepsis, immediate diagnosis and treatment are required to decrease morbidity and mortality, and improve the related therapeutic outcomes.^5^ Unfortunately, sepsis is also one of the most heterogeneous syndromes in terms of its symptomology and pathophysiology, making its diagnosis very challenging. In addition, no standard diagnostic test has been developed to detect the onset of sepsis in clinic so far.^6^ Current primary diagnostic tests based on the measurement of vital signs and scores often lack required speed or precision to make timely accurate diagnosis for early treatment.^7^ Although numerous biomarkers in serum for sepsis diagnosis have been well identified, the necessary sensitivity and reliability are still insufficient.^8^,^9^ To assess the underlying infections, microbiological tests have been proposed to aid the detection of sepsis. However, microbiological cultures usually require a long time, even a few days, for bacterial growth to provide positive results, and culture-positive “sepsis” is observed in only 30–40% of cases. In other words, sepsis is seldom confirmed microbiologically for timely medical treatment.^10^,^11^ Therefore, the development of accurate methods for early diagnosis of sepsis is urgently needed.

In the development of sepsis, the activation of the host immune system triggered by serious infection usually causes a systemic inflammation response.^12^ As a result, excessive reactive oxygen species (ROS), including hypochlorite ions (ClO^–^), hydroxyl radicals (OH·), superoxide anion radicals (O_2_·^–^), and peroxynitrite (ONOO^–^), are generated in the initial phases of sepsis.^13^–^17^ Different from local inflammation and infections during which ROS are produced in large quantities in specific inflamed tissues and organs, sepsis usually leads to elevated systemic ROS levels both in the circulation and in the affected organs.^13^ Moreover, the systemic overproduction of ROS is also a major cause of MODS during sepsis.^14^,^18^ Thus, ROS can be used as alternative and predictive indicators for sepsis, and a sensitive and timely method to monitor the systemic ROS in biological systems not only is beneficial for the early diagnosis and risk stratification of sepsis, but also helps with the research of sepsis and ROS biology.^19^,^20^

So far, molecular and nanoscale probes for sensitive ROS detection have been developed for fluorescence imaging and chemiluminescence imaging.^21^–^30^ However, during sepsis, most of the ROS are excessively generated in deep tissues and organs such as the liver and kidneys, and the unsatisfactory penetration and low soft tissue sensitivity of imaging technologies based on optics make it difficult to obtain ROS mapping with an unlimited imaging depth *in vivo* for sepsis diagnosis.^13^,^18^,^31^ Additionally, activatable photoacoustic nanoprobes have been developed for sensitive imaging of ROS, providing relatively deeper tissue penetration.^32^–^36^ However, restrictions caused by the inherent penetrability of optics have not been fundamentally resolved. By contrast, MRI which has emerged as a powerful diagnostic tool in clinic can provide high spatial resolution images of soft tissues with unlimited penetration depth.^37^–^39^

Recently, endogenous probes have been well developed to detect ROS *in vitro* and *in vivo* by using *T*_1_-weighted (T1W) MRI and QUEST MRI.^40^,^41^ These endogenous probes for ROS detection highly depend on the paramagnetism of the continuously overproduced ROS. However, different from exogenous probes which can be used to diagnose diseases by comparing signal changes before and after administration, endogenous probes are susceptible to various non-ROS factors, such as the changes in the contents of oxygen and water.^42^–^44^ Moreover, exogenous ^19^F-MRI probes have emerged as contrast agents for *in vivo* ROS imaging.^45^,^46^ However, they require the use of the ^19^F-MRI technique, which is limited by the lack of specific clinical scanners as most of them are only designed for ^1^H use.^47^ Accordingly, the rational design of novel activatable probes to detect systemic ROS for the evaluation of sepsis and the study of sepsis and related ROS biology is still highly needed.

**Fig. 1:**
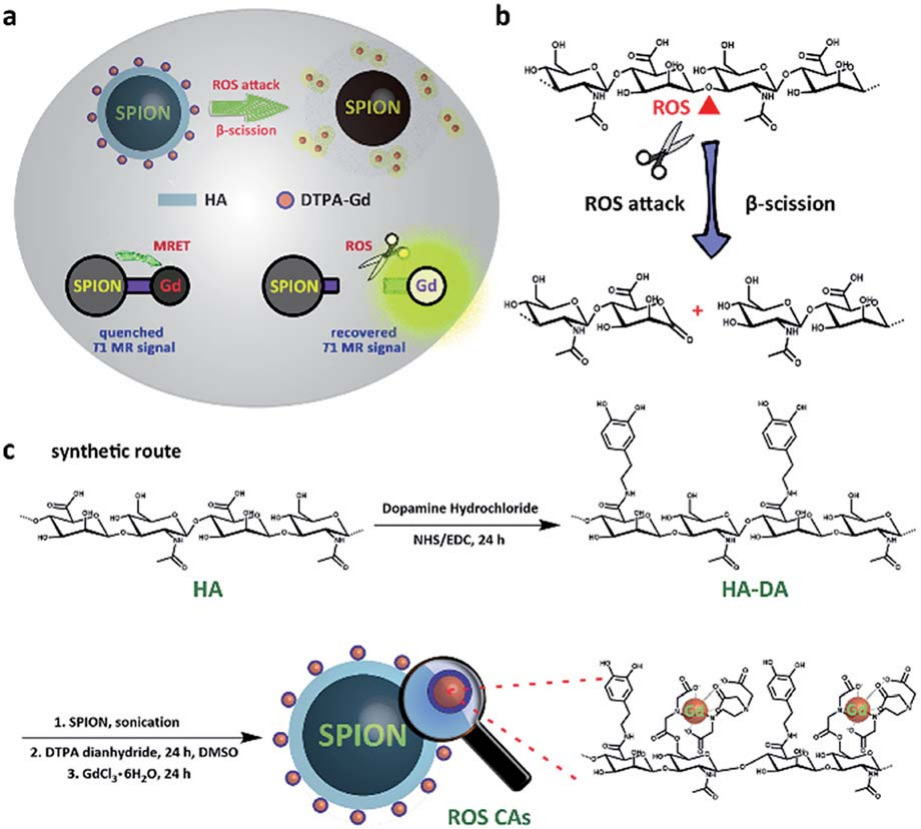
Schematic illustration of the detection mechanism of ROS CAs (a), ROS-triggered β-scission of HA (b), and rational synthesis of ROS CAs (c).

More recently, Cheon and co-workers have reported the phenomenon of distance-dependent magnetic resonance tuning that occurs between a superparamagnetic quencher and a paramagnetic enhancer.^48^–^50^ In principle, when the enhancer is localized around the quencher, the fluctuation rate of electron spin from the enhancer is decelerated, resulting in an OFF state with a quenched T1W MRI signal. In contrast, once the enhancer is separated from the quencher, the fast fluctuation rate of electron spin is recovered, leading to an ON state with a recovered T1W MRI signal. Using MRET, activatable MRI probes have been designed for pH imaging and *T*_1_–*T*_2_ dual modality imaging.^48^–^51^ Inspired by these significant findings, herein we present the rational design of novel ROS-activatable nanoprobes (ROS CAs) composed of a clinically approved iron oxide core, Gd–DTPA, and hyaluronic acid (HA) that could image ROS down to sub-micromolar concentrations *via* MRI, and use them as sensitive contrast agents for sepsis evaluation. In our design, Gd–DTPA and superparamagnetic iron oxide nanoparticles (SPION) were selected as the enhancer and the quencher, respectively. As a medicinal polysaccharide, HA acted as the linker between the quencher and the enhancer.^52^Fig. 1 illustrates the preparation of ROS CAs and the related ROS detection mechanism. Once attacked by ROS, HA backbones underwent a ROS-activated cleavage process *via* β-scission reaction, leading to the detachment of Gd–DTPA from ROS CAs and the recovery of quenched T1W MRI signals.^52^–^54^ With their outstanding sensitivity and unlimited tissue penetration depth, ROS CAs were capable of imaging the systemic ROS overproduction in mice with early sepsis. Significantly, by using ROS CAs, the severity of the sepsis could be rapidly evaluated by monitoring the ROS levels *in vivo*. We hope that the present study not only provides a new strategy to aid in the early diagnosis and risk assessment of sepsis, but also offers a valuable insight for the extensive study of sepsis and ROS biology.

## Results and discussion

**Fig. 2:**
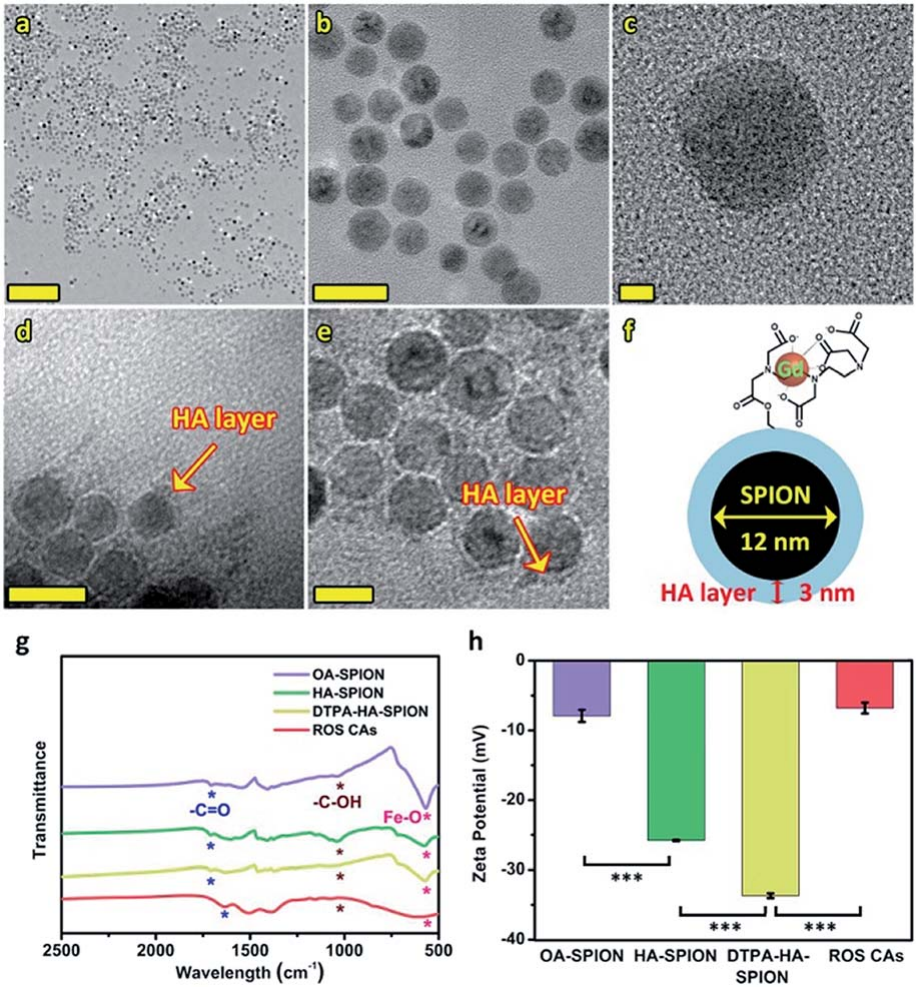
TEM images (a and b) and HR-TEM image (c) of SPION. TEM images of HA-SPION (d) and ROS CAs (e). Scale bars represent 200 nm (a), 20 nm (b), 2 nm (c), 20 nm (d), and 10 nm (e), respectively. Structural information of ROS CAs (f). FT-IR spectra (g) and *ζ* potentials (h) of OA-SPION, HA-SPION, DTPA-HA-SPION, and ROS CAs. Error bars represent standard deviation from the mean (*n* = 3). Asterisks indicate statistically significant differences (*P < 0.05, **P < 0.01, and ***P < 0.001).

Prior to the acquirement of ROS CAs, oleic acid-capped SPION (OA-SPION) were first prepared based on a classical method.^55^ The wide-angle XRD pattern of OA-SPION correlated well with the cubic spinel structure of magnetite (Fig. S1†). The TEM images and HR-TEM image demonstrate that OA-SPION possess excellent dispersity with an average diameter of 12 nm (Fig. 2a–c). To achieve ideal surface modification, HA–dopamine (HA–DA) was then synthesized *via* the well-established NHS/EDC chemistry.^56^,^57^ The UV-vis spectrum further indicated the successful formation of HA–DA and the non-oxidized state of conjugated DA (Fig. S2†). Upon a simple ultrasonic treatment, HA–DA could be attached on the surface of OA-SPION *via* the strong iron oxide–catechol bonds, thus resulting in the formation of HA capped SPION (HA-SPION).^58^ A clear HA–DA layer with a thickness of 3 nm around the magnetic cores could be visualized in the TEM image (Fig. 2d). Afterwards, DTPA dianhydride was chemically introduced into the HA backbones of HA-SPION *via* esterification reaction, and DTPA modified HA-SPION (DTPA-HA-SPION) were then formed.^59^,^60^ After the introduction of Gd^3+^, ROS CAs were finally constructed (Fig. 2e and f). FT-IR spectra, TGA data, and *ζ* potentials of various products were further explored in detail and used to confirm our design. As shown in Fig. 2g, new bands at 1050 cm^–1^ for HA-SPION could be assigned to the –C–O stretching of –CH_2_OH in HA, indicating that HA–DA was attached on the surface of magnetic cores.^61^ Compared with OA-SPION, the weakened vibration of Fe–O in HA-SPION also verified the above results. TGA data revealed that the content of HA in HA-SPION was about 46.2 wt% (Fig. S3†). Compared with HA-SPION, the formation of new ester bonds between DTPA dianhydride and HA in DTPA-HA-SPION was confirmed by the weakened –C–O stretching of –CH_2_OH groups in HA at 1050 cm^–1^. Moreover, for ROS CAs, the peak of –COOH in DTPA-HA-SPION shifted after the addition of Gd^3+^, which was ascribed to the coordination effect between –COOH and Gd^3+^. Results of *ζ* potential measurements also provided important information about the formation of these products (Fig. 2h). As expected, an obvious decrease from –7.93 mV of OA-SPION to –25.80 mV of HA-SPION successfully verified that the magnetic cores were covered with negatively charged HA. Due to the efficient attachment of carboxyl group-enriched DTPA, the *ζ* potential of DTPA-HA-SPION decreased to –33.67 mV sharply. However, after the addition of Gd^3+^, ROS CAs with a *ζ* potential of –6.8 mV were finally obtained. The above data provided detailed changes of surface functional groups and electric charge during the synthesis process of ROS CAs. Last but not least, both the TEM image and structural information of ROS CAs indicated that they were highly monodisperse with excellent size uniformity as well as their average diameter could be measured as 15 nm (Fig. 2e). ICP-MS analysis indicated that ROS CAs had a Gd content of ∼11.7 wt%. All these exciting results revealed the successful synthesis and characterization of ROS CAs.

**Fig. 3:**
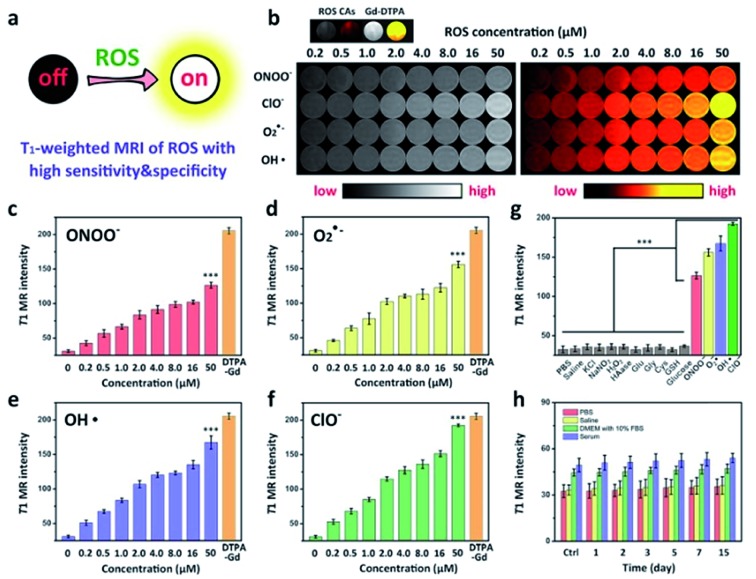
Schematic illustration of the high sensitivity and specificity of ROS CAs towards ROS (a). T1W MRI phantom images and the related colour-coded images of solution containing ROS CAs after treatment with various ROS (b). Changes in the T1W MRI signal intensity of solution containing ROS CAs after treatment with ONOO^–^ (c), O_2_·^–^ (d), OH· (e), and ClO^–^ (f). Changes in the T1W MRI signal intensity of solution containing ROS CAs and different solutes (g). Time-dependent T1W MRI signal intensity of ROS CAs after dissolving in PBS, saline, DMEM with 10% FBS, and serum (h). Error bars represent standard deviation from the mean (*n* = 3). Asterisks indicate statistically significant differences (*P < 0.05, **P < 0.01, and ***P < 0.001).

To explore the ROS-responsive properties of our ROS CAs, we first incubated them with various chemically generated ROS (Fig. 3a). Changes in T1W MRI signals were then studied on a 3.0 T MR scanner. After incubation with various sepsis-related ROS including ONOO^–^, O_2_·^–^, OH·, and ClO^–^ with a concentration of 50 μM, nearly 50% of ROS CAs were activated quickly within 5 min compared to the enhancer with the same concentration, as well as the signal intensities could reach a plateau within 30 minutes (Fig. S4†). As shown in Fig. 3b, T1W MRI signals gradually enhanced with the increasing amounts of ONOO^–^ (Fig. 3c), O_2_·^–^ (Fig. 3d), OH· (Fig. 3e), and ClO^–^ (Fig. 3f). Upon treatment with various ROS at a relatively low concentration of 0.2 μM, obvious T1W MRI signal enhancements could be detected according to T1W MRI phantom images and the related colour-coded images. Among all these ROS detections, ROS CAs showed the best sensitivity towards ClO^–^. However, we could not find any T1W MRI signal enhancement when ROS CAs were incubated with other non-strong oxidizing solutes even at a high concentration of 50 μM with 2 h of incubation (Fig. 3g). It is worth noting that H_2_O_2_ exhibited negligible influence on the MRI signal intensities due to its low cleavage ability towards HA.^52^ Moreover, hyaluronidase (HAase) showed no effect on the MRI signal intensities because the multi-step modification during the synthesis of ROS CAs might extremely hinder the identification of the HA substrate site by HAase. In addition, the stability and degradation of ROS CAs were further investigated. Results of the changes of T1W MRI signals indicated that ROS CAs exhibited admirable stability against PBS, saline (0.9% NaCl solution), DMEM with 10% FBS, and serum even when the co-incubation period was prolonged to 15 days (Fig. 3h). Taken together, all these results suggested that our ROS CAs had high sensitivity and selectivity towards various ROS.

**Fig. 4:**
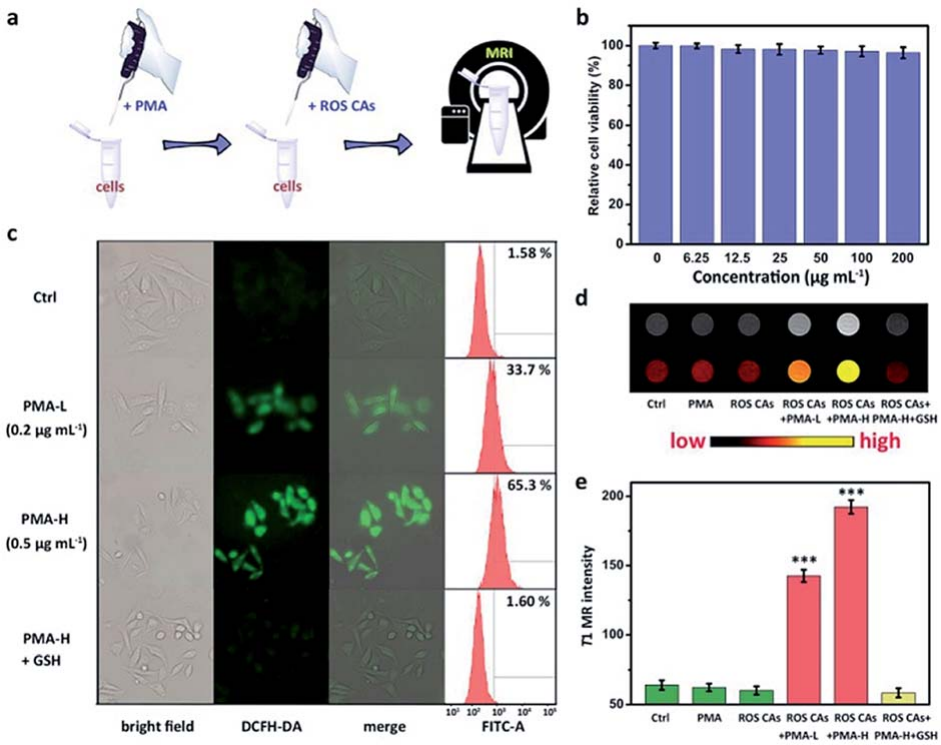
Schematic illustration of the preparation of inflammatory cells induced by PMA and related MR imaging of ROS by using ROS CAs (a). Viabilities of HeLa cells treated with ROS CAs (b). Cellular images and flow cytometry analysis of PMA-induced ROS generation in HeLa cells and related GSH treatment (c). Concentrations of PMA in the treatments of HeLa cells are described in (c). T1W MRI images of HeLa cells and related colour-coded images after different treatments (d). From left to right: cells only, cells treated with PMA, cells treated with ROS CAs, cells treated with PMA and ROS CAs, and cells pre-treated with GSH and then treated with PMA and ROS CAs. Changes in the T1W MRI signal intensity of HeLa cells after different treatments (e). Error bars represent standard deviation from the mean (*n* = 3). Asterisks indicate statistically significant differences (*P < 0.05, **P < 0.01, and ***P < 0.001).

To investigate the ROS-responsive properties of ROS CAs *in vitro*, ROS CAs were incubated with inflammatory cells (Fig. 4a). The cytotoxicity of ROS CAs was evaluated at first. As shown in Fig. 4b, an MTT assay associated with HeLa cells revealed that the viability of all the cells was not hindered by ROS CAs even at the highest concentration of 200 μg Gd per mL after 24 h of incubation. Visualized viability based on dual-staining imaging is presented in Fig. S5.† Similar to the results of the MTT assay, all the cells were alive, indicating that ROS CAs had a negligible cytotoxic effect on HeLa cells. To mimic inflammatory conditions, phorbol myristate acetate (PMA) was used here to stimulate HeLa cells to produce excess ROS.^28^ By using DCFH-DA as a typical fluorescent ROS sensor, the intracellular ROS levels could be visualized by fluorescence microscopy. As shown in Fig. 4c, cells treated with PMA revealed increased intracellular ROS levels in a dose-dependent manner, which was re-confirmed by flow cytometry analysis, suggesting that the inflammatory cell model was successfully constructed. Quantitative analysis of cellular uptake of ROS CAs was then explored by ICP-MS after cell digestion. As expected, cellular uptake of ROS CAs showed a plateau after 4 h of incubation (Fig. S6†). Thereby, 4 h was selected as the typical experimental period to image the ROS levels of inflammatory cells after the treatment with ROS CAs. Compared with the pristine cells, cells incubated with PMA and ROS CAs exhibited significantly enhanced T1W MRI signals with the increasing amounts of PMA, whereas cells treated only with ROS CAs had negligible T1W MRI signal enhancement (Fig. 4d and e).

To confirm that the T1W MRI signal enhancement was a result of the response of ROS CAs towards intracellular ROS, PMA-stimulated cells were first treated with antioxidant glutathione (GSH) before the incubation with ROS CAs. GSH is considered as a common ROS scavenger which could be used to protect cells against unwanted oxidative stress by reducing intracellular levels of ROS.^62^–^67^ Previous studies demonstrated that GSH could not only be transported into many kinds of mammalian cells, but also could be taken up by broad bean leaf tissues and protoplasts. Thus, GSH was selected as an efficient intracellular ROS scavenger in our study to confirm our design. As expected, it was easily found that pre-treatment of GSH could efficiently eliminate intracellular ROS and prevent the T1W MRI signal enhancement of inflammatory cells.

**Fig. 5:**
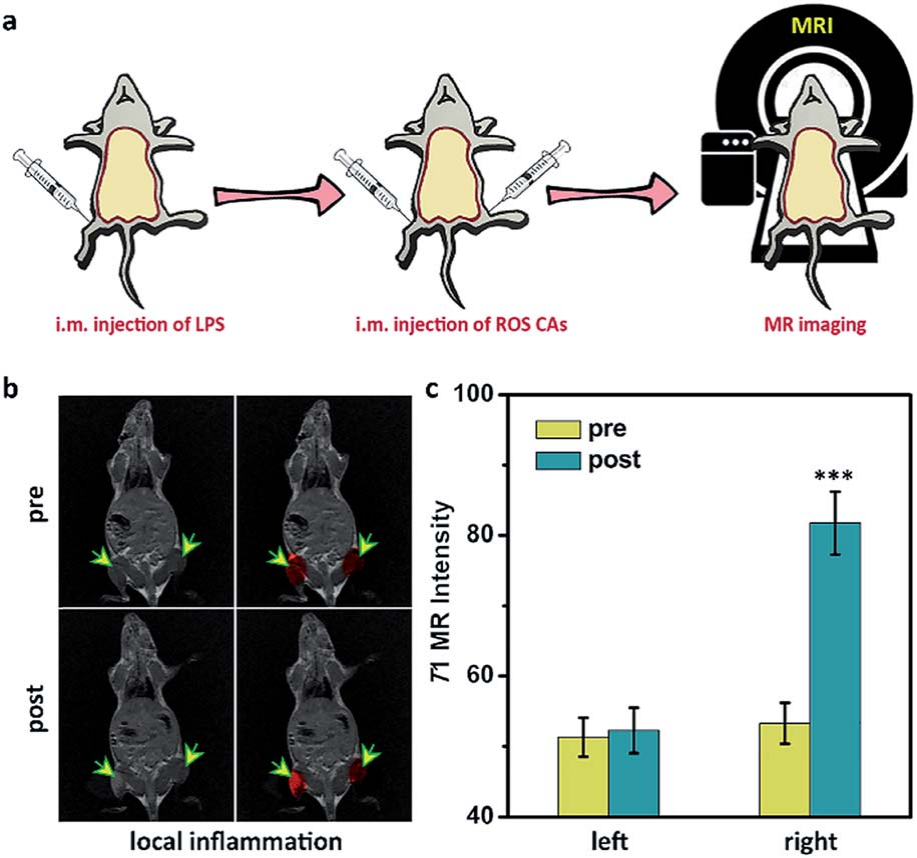
(a) Schematic illustration of the preparation of a mouse model with local inflammation and related *in vivo* inflammation imaging *via* MRI. (b) T1W MRI images of the mouse with local inflammation before and after the treatment with ROS CAs. (c) MRI intensity variation of regions of interest in a mouse after various treatments shown in (b).

**Fig. 6:**
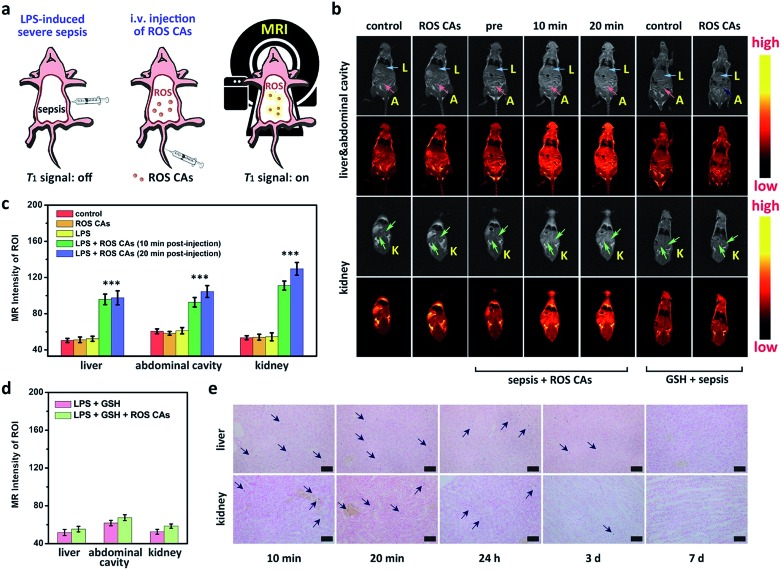
(a) Schematic illustration of the preparation of a mouse model with severe sepsis induced by LPS and related *in vivo* sepsis diagnosis using ROS CAs *via* MRI. (b) T1W MRI images of a healthy mouse, ROS CA-treated healthy mouse, septic mouse, ROS CA-treated septic mouse, GSH-treated septic mouse, and ROS CA-treated septic mouse pre-treated with GSH. Abbreviations: L for liver, A for abdominal cavity, and K for kidney. (c and d) MRI intensity variation of regions of interest in mice after various treatments shown in (b). ROI: regions of interest. Prussian staining images of the liver and kidneys in septic mice (e). Scale bars represent 50 μm. Error bars represent standard deviation from the mean (*n* = 3). Asterisks indicate statistically significant differences (*P < 0.05, **P < 0.01, and ***P < 0.001).

After understanding the *in vitro* performance of ROS CAs, their feasibility for *in vivo* imaging of ROS was evaluated by using a local inflammation mouse model after intramuscular (i.m.) injection of lipopolysaccharide (LPS). As shown in Fig. 5, compared with the sites without any treatment and simple inflammatory site, higher MR intensity from the inflammatory site after the intramuscular injection of ROS CAs could be easily detected based on the *in vivo* T1W MRI, indicating that the local inflammatory site could be well visualized by using our ROS CAs. We then sought to use ROS CAs to track sepsis *in vivo*. Firstly, mice were intraperitoneally injected with a high dose of LPS, and a mouse model of severe sepsis was established 6 h post-injection (Fig. 6a). As expected, LPS-treated mice showed a significant increase in alanine transaminase (ALT), interleukin-6 (IL-6), and tumour necrosis factor-α (TNF-α) levels in serum, indicating the successful construction of an LPS-induced sepsis mouse model (Fig. S7–S9†).^4^,^31^,^68^ Afterwards, mice were intravenously injected (i.v.) with ROS CAs and imaged on a 3.0 T MR scanner. Representative T1W MRI images centred on the liver, kidneys, and abdominal cavity are described in Fig. 6b. Compared with the healthy mice, mice with severe sepsis exhibited no T1W MRI signal intensity changes. However, owing to the efficient separation of the quencher (SPION) and the enhancer (Gd–DTPA) caused by the ROS-triggered degradation of the HA linker, an obvious positive T1W MR contrast could be visualized throughout the bodies of the septic mice treated with ROS CAs, demonstrating that our well-designed activatable nanoprobes could act as sensitive reporters of sepsis *in vivo*. Specifically, the T1W MRI signal intensities of the septic mice were nearly doubled in the liver, kidneys and abdominal cavity 10 min post-injection of ROS CAs, relative to the signal intensities before injection (Fig. 6c). Moreover, the above obviously enhanced T1W MRI signal triggered by ROS could last for more than 20 min, which might be caused by the continuous ROS-activated cleavage process of the HA linker. To re-confirm that the T1W MRI signal enhancement was a result of the response of ROS CAs towards sepsis, septic mice were intravenously injected with GSH before the injection of ROS CAs. It was easily found that the administration of GSH could efficiently block the T1W MRI signal enhancement of the septic mice, indicating that ROS CAs could specifically image systemic ROS for sepsis diagnosis (Fig. 6b and d). Moreover, luminol L-012 was selected and used as a typical chemiluminescence sensor to image ROS *ex vivo*. As shown in Fig. S10,† there was indeed a large amount of ROS produced in the liver and kidneys of septic mice. However, compared with septic mice, nearly no luminescence signal could be found in healthy mice. These results re-confirmed the feasibility of our ROS CAs in detecting ROS by using the MRI technique and the successful establishment of LPS-induced sepsis. Afterwards, the peritoneal fluid and blood from septic mice were collected and imaged *ex vivo*. As expected, the peritoneal fluid from septic mice brightened 10 min and 20 min post-injection of ROS CAs, indicating the overproduction of ROS in peritoneal fluid (Fig. S11a†). These ROS in peritoneal fluid were generated from infiltrated neutrophils and macrophages caused by the septic peritonitis. However, the blood of septic mice did not exhibit obvious T1W MRI signal enhancement (Fig. S11b†). These results thus indicated that the content of ROS in blood was relatively low even in the case of severe sepsis during the whole imaging period. Taking the results together, we concluded that the whole body signal elevation in the LPS-treated mice could be attributed to the sepsis-induced systemic inflammation rather than the circulating released enhancer owing to the strong buffering capacity of blood. These exciting results suggested that our ROS CAs with an admirable ability of great response towards systemic elevated ROS could be used for sepsis diagnosis *in vivo*.

Bio-distributions of SPION and Gd^3+^ in septic mice post-injection of ROS CAs were explored by Prussian blue staining and ICP-MS. Single-dose injection of ROS CAs was used all through the bio-distribution investigation. As shown in Fig. 6e, Prussian blue staining images indicated that a large number of SPION were accumulated in the liver and kidneys of septic mice post-injection of ROS CAs within a relatively short period. With the passing of time, these probes could be eliminated from the mouse’s body 7 days after the intravenous injection. Moreover, the ICP-MS results showed that a considerable amount of Gd^3+^ was accumulated in the liver and kidneys of septic mice post-injection of ROS CAs within the first 20 min (Fig. S12†). However, due to the relatively short blood circulation time of Gd–DTPA, it could be rapidly excreted from the mouse’s body after the ROS-triggered HA degradation. It was worth noting that the released enhancer from ROS CAs could be excreted within 24 h whether in the early or severe sepsis, thus minimizing the chance that the residual agent would interfere with the next diagnostic tests. More importantly, the condition that nearly no Gd^3+^ was detected 24 h after the single-dose injection of ROS CAs in septic mice also demonstrated that our ROS CAs had a fast-response property towards ROS *in vivo*. Fig. 3h indicated that ROS CAs showed excellent stability in various physiological solutions. Thus, we further explored the stability and bio-distribution of ROS CAs in healthy mice. Different from the removal manner of Gd^3+^ in septic mice, Fig. S12† revealed that Gd^3+^ in healthy mice was not rapidly eliminated from the mouse’s body within the first 24 h because ROS CAs with high stability were not degraded in the absence of ROS. All these results indicated the excellent ROS-response property of ROS CAs and their potential for clinical translation.

**Fig. 7:**
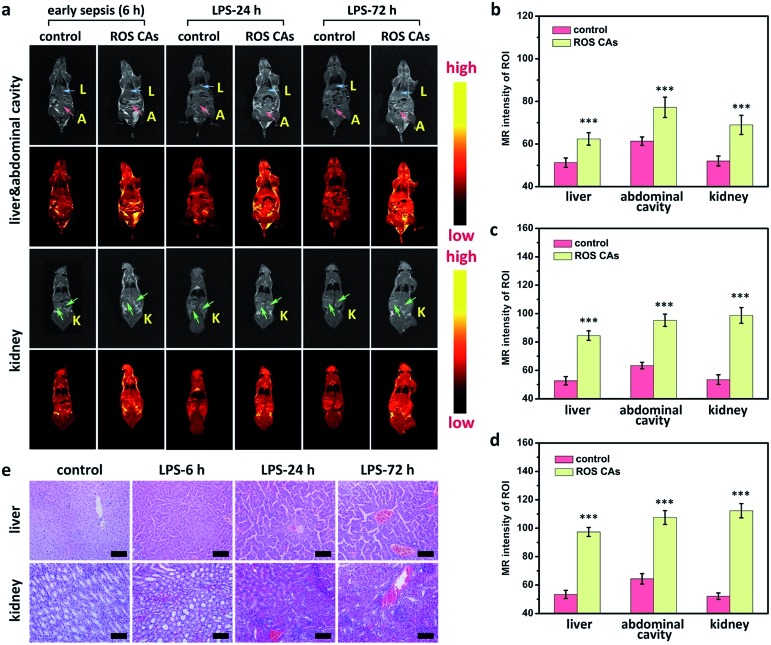
*In vivo* sepsis diagnosis using ROS CAs at different time points post-injection of 0.1 mg kg^–1^ LPS (a). Abbreviations: L for liver, A for abdominal cavity, and K for kidney. (b) MRI intensity variation of regions of interest in mice 6 h post-injection of LPS (b), 24 h post-injection of LPS (c), and 72 h post-injection of LPS (d). ROI: regions of interest. H&E staining images of the liver and kidneys in septic mice (e). Scale bars represent 50 μm. Error bars represent standard deviation from the mean (*n* = 3). Asterisks indicate statistically significant differences (*P < 0.05, **P < 0.01, and ***P < 0.001).

A critical unmet need in combating sepsis in clinic is the lack of early biomarkers with admirable reliability.^2^ Encouraged by the satisfactory sensitivity of ROS CAs, we attempted to use ROS CAs for imaging early sepsis and its progression. A mouse model with early sepsis was established by intraperitoneally injecting a low dose of LPS (0.1 mg kg^–1^), and the successful construction of the mouse model with early sepsis was verified by the moderate increase in ALT, IL-6, and TNF-α levels in serum 6 h post-injection of LPS (Fig. S13†).^69^,^70^ Then, mice were intravenously injected with ROS CAs and imaged on a 3.0 T MR scanner. As shown in Fig. 7a, mice with early sepsis treated with ROS CAs revealed an increased T1W MRI signal in the liver, kidneys, and abdominal cavity. Quantification of MRI signals further demonstrated that early sepsis could be imaged by ROS CAs (Fig. 7b). Notably, compared with mice suffering from severe sepsis with dramatically increased systemic ROS levels, the signal enhancement in mice with early sepsis was relatively low when using ROS CAs as imaging probes. During the course of sepsis progression from early sepsis to severe sepsis, ROS levels caused by the inflammation response could gradually increase. Thus, we further explored the possibility of using ROS CAs as tracers towards the development of sepsis. At different time points post-injection of LPS, mice were intravenously injected with ROS CAs and imaged on a 3.0 T MR scanner. Indeed, the MRI signal intensities gradually increased in the liver, kidneys, and abdominal cavity from 6 h to 72 h following the injection of LPS (Fig. 7a). Quantification of MRI signals further indicated the gradually enhanced systemic inflammation imaged by ROS CAs (Fig. 7b–d). The progression of sepsis in mice was further verified by the serum levels of ALT, IL-6, and TNF-α (Fig. S13†). In addition, *ex vivo* histopathological analysis was used to assess the inflammation status of the septic mice during the development of sepsis. As shown in Fig. 7e, H&E staining images indicated the progression of injury in both liver and kidneys during the development of sepsis. These results thus demonstrated that ROS CAs could diagnose early sepsis with fast response and evaluate the severity of sepsis for better risk assessment.

Finally, we explored the long-term toxicity of ROS CAs after intravenous injection. Blood biochemistry and haematology were firstly used to provide a quantitative assessment of long-term toxicity of ROS CAs. As shown in Fig. S14,† there were no significant differences between the test group and the control group, and all the parameters fell well in the reference index. Moreover, at the end of different experimental periods, no injury of major organs from mice treated with ROS CAs could be detected based on the H&E staining images and all the images showed a similar pathological structure to that from the healthy mice (Fig. S15†). Last but not least, mouse body weight measurements and behaviour observation were performed to explore the potential toxicity of ROS CAs after intravenous injection. Compared with the control group, mice in the test group exhibited negligible differences in body weight, eating, drinking, activity, and neurological status during the whole experimental period (Fig. S16†). All these results suggested the overall safety of ROS CAs, which were formed with three kinds of clinically approved reagents.

## Conclusions

In summary, we reported the rational construction of novel ROS CAs for MRI of ROS levels *in vivo*, and further used them as sensitive contrast agents for sepsis evaluation. Our well-designed nanoprobes were composed of clinically approved SPION, Gd–DTPA, and HA. These well-prepared ROS CAs were highly sensitive towards various ROS including ONOO^–^, ClO^–^, O_2_·^–^, and OH· that were systemically over-produced during sepsis. Results based on both *in vitro* and *in vivo* experiments demonstrated that ROS CAs could not only image ROS with unlimited tissue penetration depth for early sepsis evaluation, but also precisely track systemic ROS to evaluate the severity of sepsis for better risk assessment. We believe that our study could provide a new strategy for early diagnosis of sepsis with fast response and facilitate the design of novel ROS probes for deep tissue use.

## Ethical statement

All animal experiments were performed in accordance with the NIH guidelines for the care and use of laboratory animals (NIH Publication no. 85-23 Rev. 1985) and approved by the Jilin University Animal Care and Use Committee. Balb/c mice (8–10 weeks, 25 g) were obtained from the Laboratory Animal Center of Jilin University (Changchun, China), and all animal care and handling procedures were in accordance with the guidelines approved by the ethics committee of Jilin University.

## Conflicts of interest

The authors declare no competing interests.

## Supplementary Material

Supplementary information
