# Supplementary material for: Ultrasensitive magnetic resonance imaging of systemic reactive oxygen species in vivo for early diagnosis of sepsis using activatable nanoprobes
Source: Chem Sci. 2019 Feb 21;10(13):3770–8. doi: 10.1039/c8sc04961k (PMC6447818; doi:10.1039/c8sc04961k)
Supplement: Supplementary file 1 [file SC-010-C8SC04961K-s001.pdf]

Electronic Supplementary Material (ESI) for Chemical Science.
This journal is © The Royal Society of Chemistry 2019

Supporting Information

Ultrasensitive Magnetic Resonance Imaging of Systemic Reactive Oxygen Species *In Vivo* for Early Diagnosis of Sepsis Using Activatable Nanoprobes

Huan Wang,[a,b] Dongqin Yu,[a,b] Bo Li,[c] Zhen Liu,*[,a,d] Jinsong Ren,*[,a] and Xiaogang Qu*[,a]

a. State Key Laboratory of Rare Earth Resources Utilization and Laboratory of Chemical Biology, Changchun Institute of Applied Chemistry, Chinese Academy of Sciences, Changchun, Jilin, 130022, P. R. China

b. University of Science and Technology of China, Hefei, Anhui, 230029, P. R. China

c. Department of Radiology, The Second Hospital of Jilin University, Changchun, Jilin, 130041, P. R. China

d. Beijing Advanced Innovation Center for Soft Matter Science and Engineering, Beijing University of Chemical Technology, Beijing 100029, P. R. China

Ⅰ. Experimental Section

Chemicals:

$FeCl_3·6H_2O$, $GdCl_3·6H_2O$, oleic acid, 1-octadecene, calcein AM, propidium iodide (PI), 2-(N-morpholino) ethanesulfonic acid (MES), 1-ethyl-3(3-dimethylaminopropyl) carbodiimide (EDC), N-hydroxysuccinimide (NHS), glutathione (GSH), phorbol myristate acetate (PMA) and lipopolysaccharide (LPS) were purchased from Sigma-Aldrich. Sodium oleate and diethylenetriaminepentaacetic dianhydride (DTPA dianhydride) were purchased from TCI. Hyaluronic acid (HA, 10 kDa) and dopamine hydrochloride were purchased from Aladdin Reagent. Ultrapure water was prepared using Milli-Q-Plus water system (18.2 MΩ cm^{-1}) and used in all the experiments. All other reagents were of analytical grade and used as received without any purification.

Synthesis of superparamagnetic iron oxide nanoparticles (SPION):

SPION with an average diameter of 12 nm were synthesized according to a classical method. Briefly, $FeCl_3·6H_2O$ (40 mmol) and sodium oleate (120 mmol) was dissolved in a mixture solvent containing ethanol (80 mL), ultrapure water (60 mL), and hexane (140 mL). The resulting solution was heated to 70 °C and kept at this temperature for 4 h. Later, upper organic layer containing iron-oleate complex was washed with ultrapure water. After hexane was evaporated off, iron-oleate complex was obtained in a waxy solid form. Then, iron-oleate complex (40 mmol) and oleic acid (20 mmol) were dissolved in 1-octadecene (200 g) at room temperature. The reaction mixture was heated to 320 °C with a heating rate of 3.3 °C min^{-1} and kept at this temperature for 0.5 h. The resulting solution containing SPION was cooled to room temperature naturally, and ethanol (500 mL) was added to the solution to precipitate the nanoparticles. Finally, the nanoparticles were separated via centrifugation.

Synthesis of HA-dopamine (HA-DA):

HA-dopamine was prepared via classical NHS/EDC coupling reaction. HA (200 mg) was dissolved in degassed MES buffer (pH = 6, 20 mM, 50 mL). EDC (100 mg) and NHS (60 mg) were then added to this

solution and stirred for another 0.5 h. Dopamine hydrochloride (100 mg) was subsequently added. After stirring for 24 h at room temperature, solution was purified via dialysis and further freeze-dried.

Synthesis of HA-DA-SPION:

HA-DA-SPION were prepared via the strong interaction between iron oxide and catechol bonds. Typically, HA-DA (200 mg) was dissolved in ultrapure water (50 mL), and SPION (20 mg) were subsequently added. After ultrasonic treatment for 2 h, unreacted reagent was removed by centrifugation. Then, HA-DA-SPION were achieved and re-dispersed in water.

Synthesis of DTPA-HA-DA-SPION:

DTPA-HA-DA-SPION were prepared via an esterification reaction. HA-DA-SPION (20 mg) and DTPA dianhydride (200 mg) were dispersed in dry DMSO (30 mL), and the solution was stirred vigorously for 24 h. Then, ultrapure water (30 mL) was added to the mixture within 0.5 h under stirring. Unreacted reagent was removed by centrifugation and the resulting products were obtained after careful washing.

Synthesis of ROS CAs:

ROS CAs were prepared via the interaction between DTPA and Gd^{3+}. DTPA-HA-DA-SPION (20 mg) and $GdCl_3 \cdot 6H_2O$ (10 mg) were dispersed in ultrapure water (30 mL), titrated to a neutral status, and stirred to obtain stable Gd-DTPA chelation. Then, ROS CAs were achieved and washed with ultrapure water.

Characterization:

FT-IR was carried out on a BRUKER Vertex 70 FT-IR spectrometer. UV-vis spectra were performed on a JASCO V-550 Ultraviolete-visible spectrophotometer. TEM images were recorded on a FEI TECNAI G2 20 high-resolution transmission electron microscope. The ζ potential measurement was carried out on Malvern Nano ZS-90. Crystalline structures of the as-prepared samples were evaluated by X-ray diffraction analysis on a Rigaku-Dmax 2500 diffractometer. ICP-MS was carried out on an Agilent 7700x series ICP-MS instrument.

Stability of ROS CAs:

ROS CAs were incubated with PBS, saline, DMEM with 10% FBS, and serum. Then, MRI phantom T1W images were recorded on a 3.0 T MR scanner (Siemens Magnetom Avanto) at different time points. Relative MR signal intensity was further Quantificationally measured via Image J software.

Chemically generated reactive oxygen species (ROS):

$ClO^-$and $O_2^{\cdot -}$ stock solutions were prepared by diluting commercial NaClO and KO_2, respectively. OH· was generated *via* Fenton reaction from H_2O_2 reacted with $CuSO_4$ at a molar ratio of 10:1. $ONOO^-$ was obtained by H_2O_2 reacted with $NaNO_2$ at a molar ratio of 5:1.

MTT assay:

Hela cells with a density of 10^4 were seeded in a 96-well plate and further incubated in DMEM containing FBS (10%) for 12 h at 37 °C. ROS CAs with various concentrations were then added and cells were incubated for another 24 h. After incubation, medium was removed, and MTT was added. 4 h later, formazan crystals were lysed by the addition of DMSO. Bio-Rad model-680 microplate reader was used to measure the absorbance at 490 nm with 630 nm as reference.

In vitro inflammatory model:

Hela cells with a density of 10^5 were seeded in a 6-well plate for 12 h at 37 °C. PMA were added and incubated for another 1 h. Then, cells were washed with 0.9% NaCl solution and incubated with DCFH-DA. ROS fluorescence intensity of above treated cells was monitored via flow cytometry and Olympus BX-51 optical system.

Cellular uptake:

Hela cells with a density of 10^5 were seeded in a 6-well plate. After 24 h attachment, cells were treated with ROS CAs (0.1 mM) for different periods. Then, cells were washed with 0.9% NaCl solution and trypsinized. After centrifugation, cells were washed, re-suspended, and counted. Later, cells were dissolved in HNO_3 at 80 °C for 2 h, and Gd amounts in above samples were explored via ICP-MS.

Cellular viability observation:

Hela cells with a density of 2×10^4 were plated in a 12-well plate for 4 h to allow the attachment. After cells were washed twice by 0.9% NaCl solution, ROS CAs with various concentrations were added to the medium. 24 h later, cells were washed to remove the remaining nanoparticles, stained with calcein AM and PI, as well as observed under an Olympus BX-51 optical system.

ROS-responsive properties of ROS CAs:

To evaluate their ROS-responsive properties, ROS CAs (Gd: 0.1 mM) were incubated with various ROS at 37 °C. After incubation, MRI phantom T1W images were measured and recorded on a 3.0 T MR scanner at different time points. To investigate the selectivity, ROS CAs were incubated with different solutes (50 μM) at 37 °C for 2 h, and MR phantom T1W images were measured and recorded. Quantificationally, MR signal intensity was measured via Image J software.

MR imaging of ROS in vitro:

Hela cells with a density of 2×10^4 were seeded in a 24-well plate for 12 h. For *in vitro* ROS imaging, cells were treated with PMA for 1 h before the incubation with ROS CAs (Gd: 0.1 mM), which was defined as the positive group. As a comparison, PMA-treated cells were incubated with GSH (3 mg mL^{-1}) before their further incubation with ROS CAs. 4 h later, all the MRI phantom images were measured and recorded on a 3.0 T MR scanner. Relative MR signal intensity was quantificationally measured via Image J software.

Animals:

Balb/c mice (8-10 week, 25 g) were obtained from Laboratory Animal Center of Jilin University (Changchun, China), and all study protocols involving animals were approved by the Jilin University Animal Care and Use Committee. All animal care and handing procedures were in accordance with the guidelines approved by the ethics committee of Jilin University.

Local inflammation diagnosis using ROS CAs:

Mouse model with local inflammation was developed by using LPS-treated Balb/c mice as typical experimental animals. LPS was intramuscularly injected (0.1 mg kg^{-1}) at first. 24 h later, mice were

anesthetized via chloral hydrate (10% w/w) and ROS CAs were intramuscularly injected at the same time. MR imaging was obtained on a 3.0 T MR scanner 10 min after the injection of ROS CAs. Relative signal intensity was measured via Image J software.

Sepsis diagnosis in vivo using ROS CAs:

Sepsis model was developed by using LPS-treated Balb/c mice as typical experimental animals. LPS was intraperitoneally injected (1 mg kg^{-1}). Mice were anesthetized via chloral hydrate (10% w/w) 6 h after the injection of LPS, and ROS CAs (2 mg Gd kg^{-1}) were further intravenously injected. Mice without LPS injection were defined as control. MR imaging was obtained on a 3.0 T MR scanner 10 min and 20 min later after the injection of ROS CAs. Relative signal intensity was measured via Image J software. In addition, sepsis mice were intravenously injected with GSH (2 g kg^{-1}) 0.5 h before the injection of ROS CAs, which was used to prove the true source of the increased T1 contrast.

Early sepsis diagnosis in vivo using ROS CAs:

For early sepsis, LPS with a relatively low concentration (0.1 mg kg^{-1}) was intraperitoneally injected at first. Then, mice were anesthetized via chloral hydrate (10% w/w) 6 h post-injection of LPS, and ROS CAs (2 mg Gd kg^{-1}) were intravenously injected. MR imaging were obtained on a 3.0 T MR scanner 10 minutes later. To investigate whether ROS CAs could be used to track the development of sepsis, ROS CAs (2 mg Gd kg^{-1}) were also administered at 24 h and 72 h post-injection of LPS. All the signal intensities were measured via Image J software. Moreover, above mice were sacrificed, and relative inflammatory organs were harvested for essential histological analysis.

Ex vivo imaging of ROS in peritoneal fluid and blood using ROS CAs:

LPS (1 mg kg^{-1}) was intraperitoneally injected at first. Then, ROS CAs (2 mg Gd kg^{-1}) were intravenously administered 6 h later. 10 min or 20 min later, peritoneal fluid and blood was collected. MR imaging were obtained on a 3.0 T MR scanner and relative signal intensity was measured via Image J software.

Serum test:

Mice treated with LPS or 0.9% NaCl solution were sacrificed at each expected time point. Levels of ALT in serum were then explored and analyzed. Moreover, the levels of TNF-α and IL-6 in serum were analyzed by using commercial ELISA kits.

Hematology analysis and blood biochemical assay:

Blood of healthy mice treated with or without ROS CAs (2 mg Gd kg^{-1}) were collected at each expected time point, and relative samples were used to perform hematological analysis and blood biochemical assay.

Histological analysis:

Mice were sacrificed 7 d/30 d after the intravenous injection with or without ROS CAs (2 mg Gd kg^{-1}) , and main organs were then harvested, fixed with paraformaldehyde (4%), dehydeated, embedded in paraffin, sectioned, as well as stained with hematoxylin and eosin (H&E).

Bio-distribution Studies:

For bio-distribution studies, healthy mice and mice treated with LPS were intravenously injected with ROS CAs (2 mg Gd kg^{-1}) 6 h post-injection of LPS and sacrificed at each expected time point. Liver and kidneys were collected for the quantitative analysis of the bio-distribution of ROS CAs. Typically, organs were surgically removed from the mice and dissolved in aqua regia. The mixtures were heated at 80 °C for 2 h, and the Gd amounts (%ID/g) in above samples were explored by using ICP-MS.

Ex vivo imaging of ROS using Luminol-L012:

Healthy mice and LPS-treated mice were firstly anesthetized with chloral hydrate (10% w/w) and intravenously injected with L-012 (Wako, 200 mg kg^{-1}). Then, mice were scarified, and main organs were collected and imaged using an IVIS Spectrum 200. Relative images were analyzed using Image J Software.

Prussian blue staining:

Harvested tissues were fixed with paraformaldehyde (4%), dehydeated, embedded in paraffin, sectioned, and stained with Prussian blue kits (Solarbio, Beijing, China). Slides were observed on an Olympus BX-51 optical system.

Body-weight measurement:

For *in vivo* body-weight measurements, two groups of Balb/c mice were randomly separated at first. ROS CAs (2 mg Gd kg^{-1}) and 0.9% NaCl solution with the same volume were intravenously injected into the mice. Mouse body weight was recorded for 30 d.

Statistical analysis:

All data were expressed as mean result ± standard deviation (SD) based on at least three independent experiments. A p-value <0.05 was considered statistically significant. Statistical analysis was performed by using Origin 8.0 software.

Ⅱ. Supporting Figures

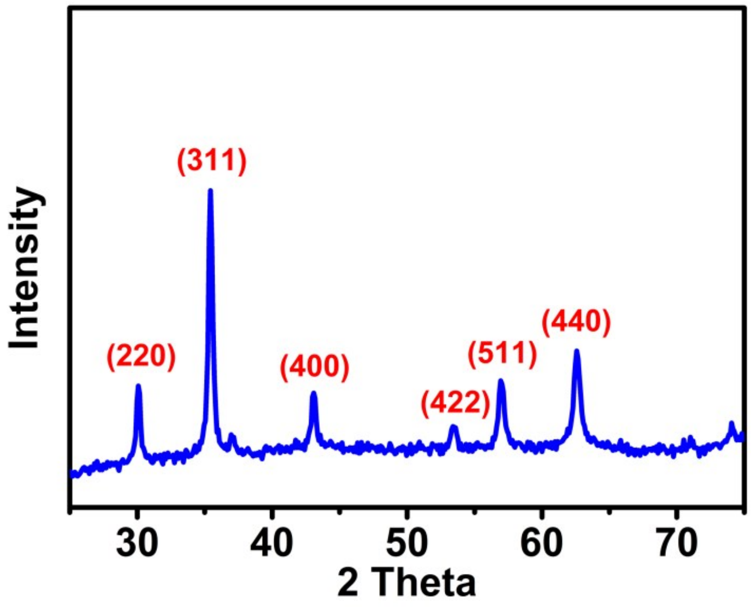

Fig. S1. Wide-angle XRD pattern of SPION.

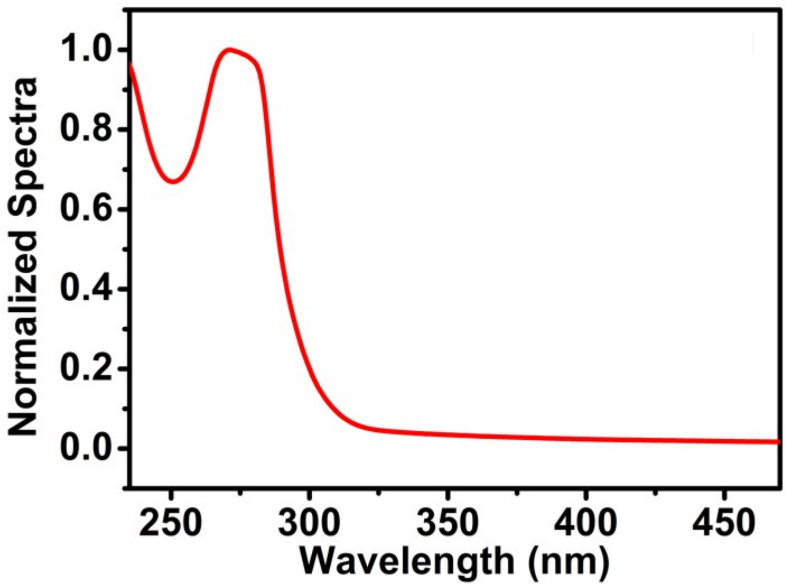

Fig. S2. UV-vis spectra of HA-DA.

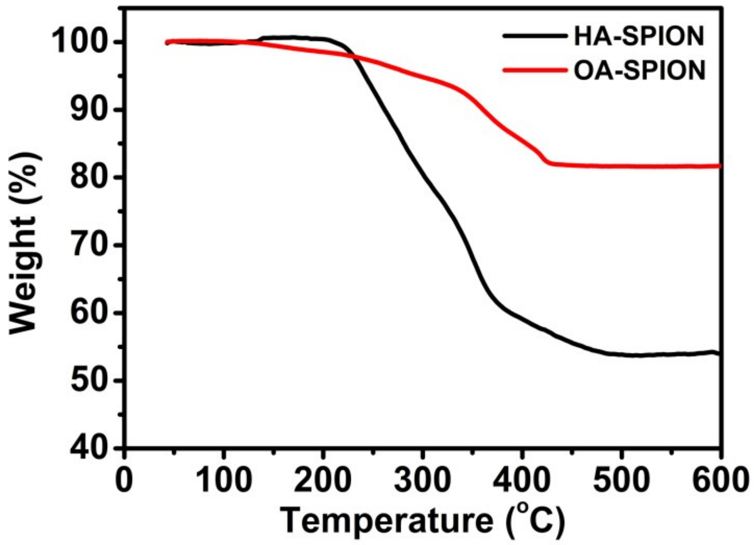

Fig. S3. Thermogravimetric analysis (TGA) of OA-SPION and HA-SPION in N_2 atmosphere.

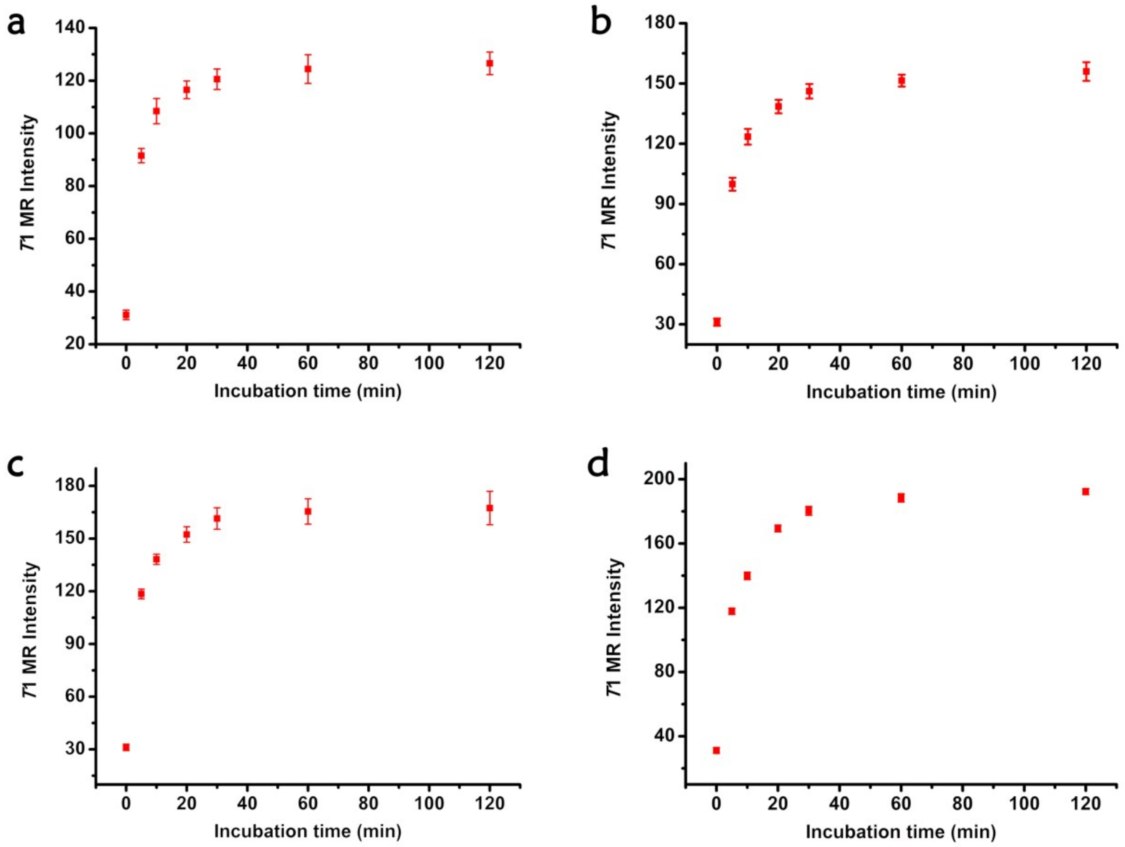

Fig. S4. Dynamics investigation of T1W MRI signal enhancement triggered by $ONOO^-$ (a), $O_2{\bullet}^-$ (b), OH• (c), and ClO^- (d).

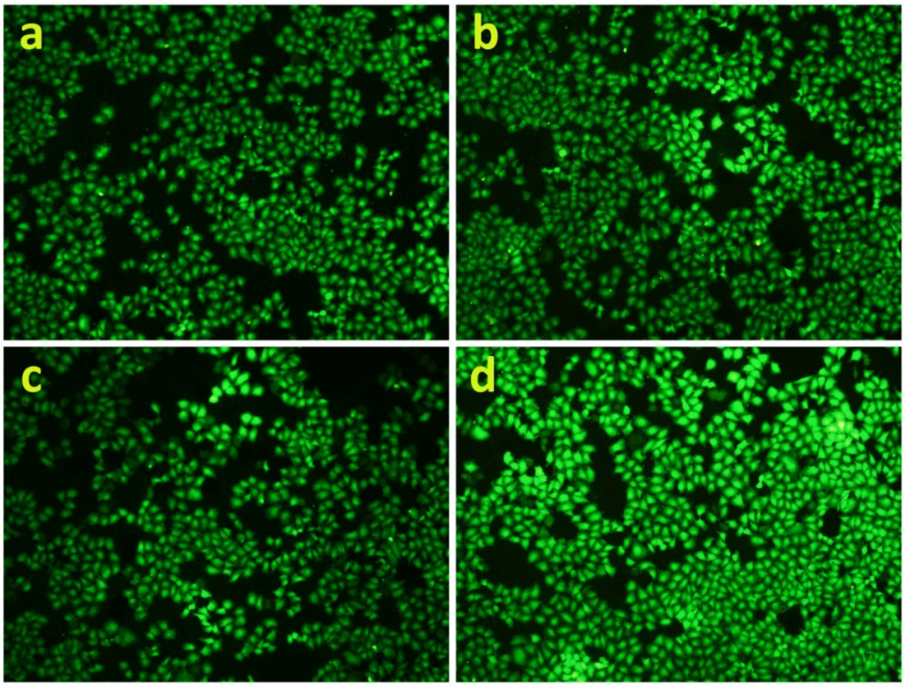

Fig. S5. Images of Calcein AM/PI dual stained cells. Hela cells were observed under a fluorescence microscope at 200×magnification. Cells were treated with 0 μg Gd mL^{-1} (a), 50 μg Gd mL^{-1} (b), 100 μg Gd mL^{-1} (c), and 200 μg Gd mL^{-1} (d) of ROS CAs.

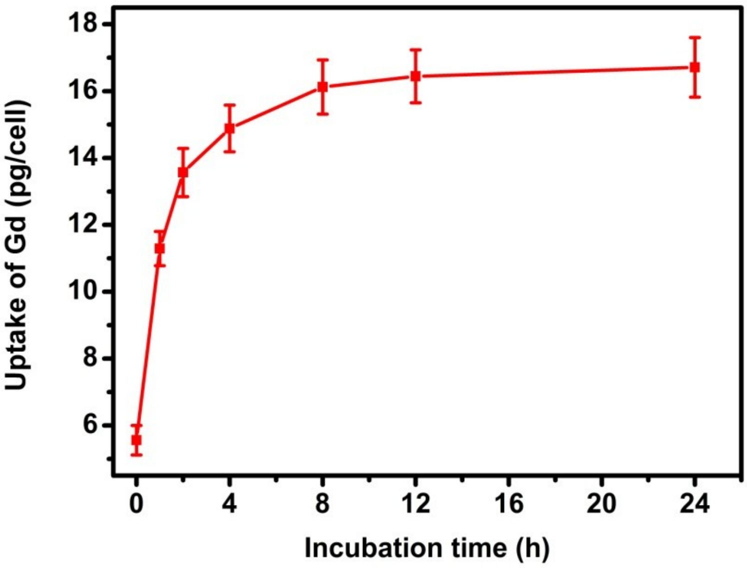

Fig. S6. Cellular uptake of ROS CAs by Hela cells at different time points.

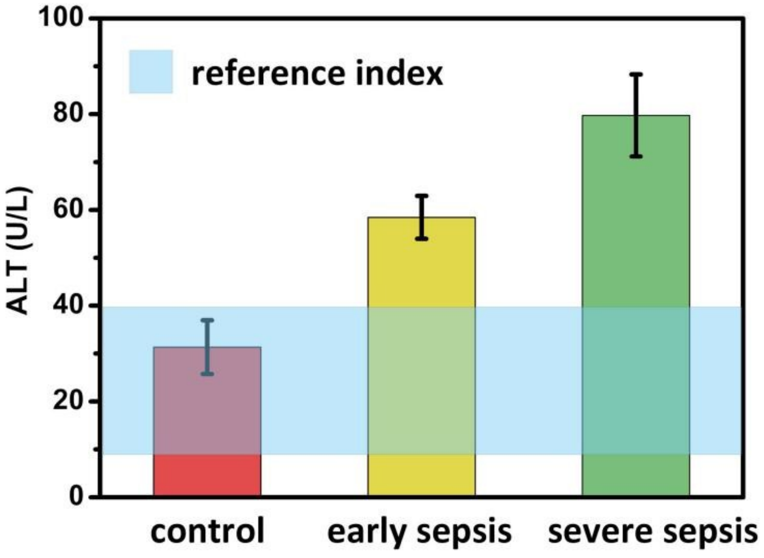

Fig. S7. ALT levels in serum of mice with severe sepsis and early sepsis.

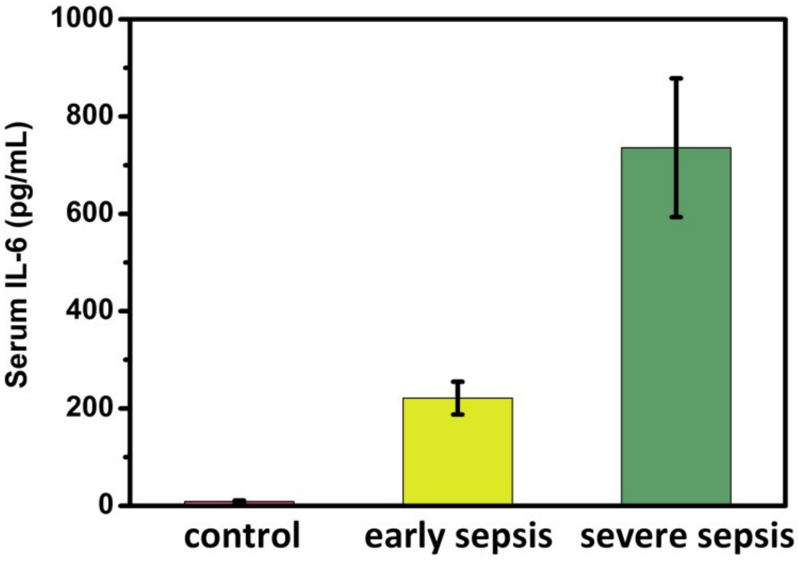

Fig. S8. IL-6 levels in serum of mice with severe sepsis and early sepsis.

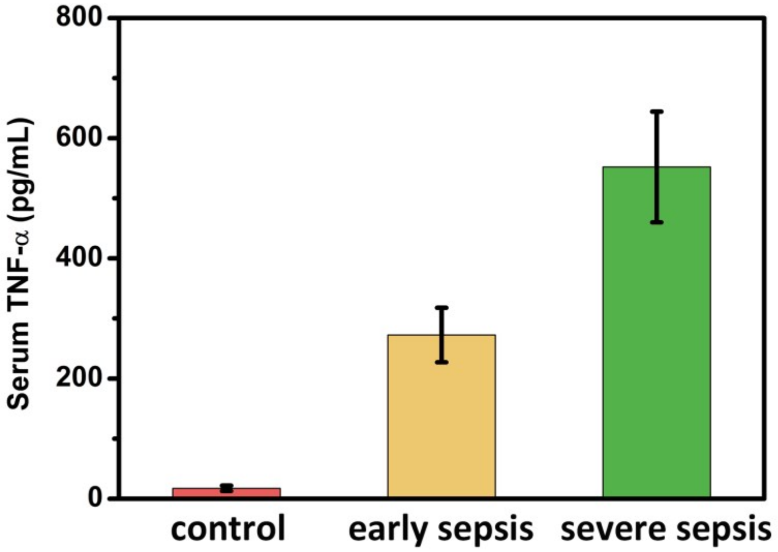

Fig. S9. TNF-α levels in serum of mice with severe sepsis and early sepsis.

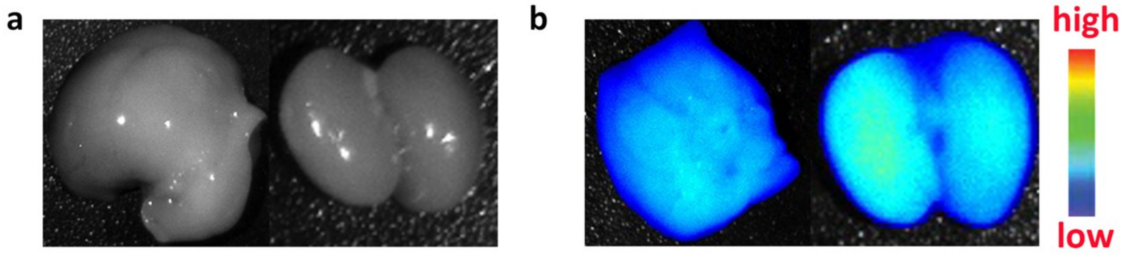

Fig. S10. *Ex vivo* imaging of ROS using Luminol-L012. Chemiluminescence images of liver and kidneys of healthy mouse (a) and septic mouse (b) post-injection of L-012, respectively.

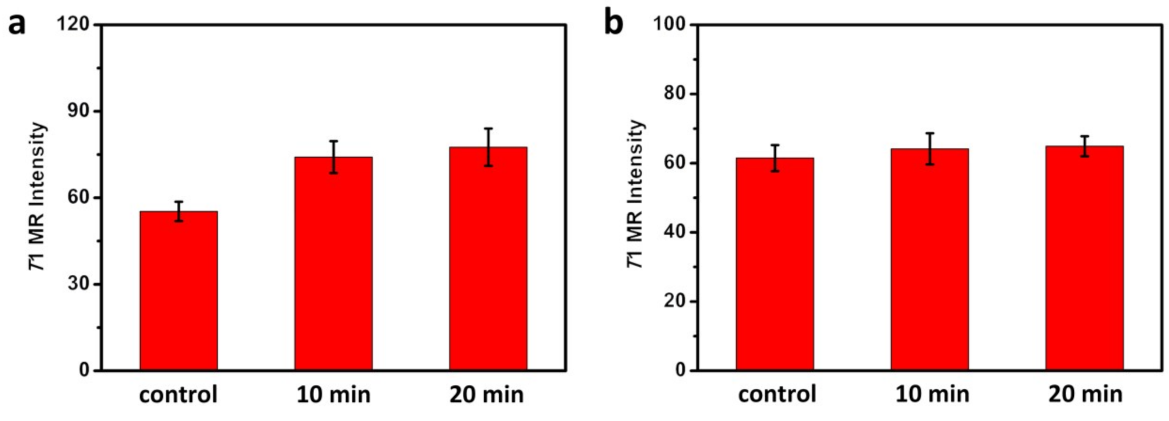

Fig. S11. Changes in T1W MRI signal intensity of peritoneal fluid (a) and blood (b) from septic mice 10 min and 20 min post-injection of ROS CAs.

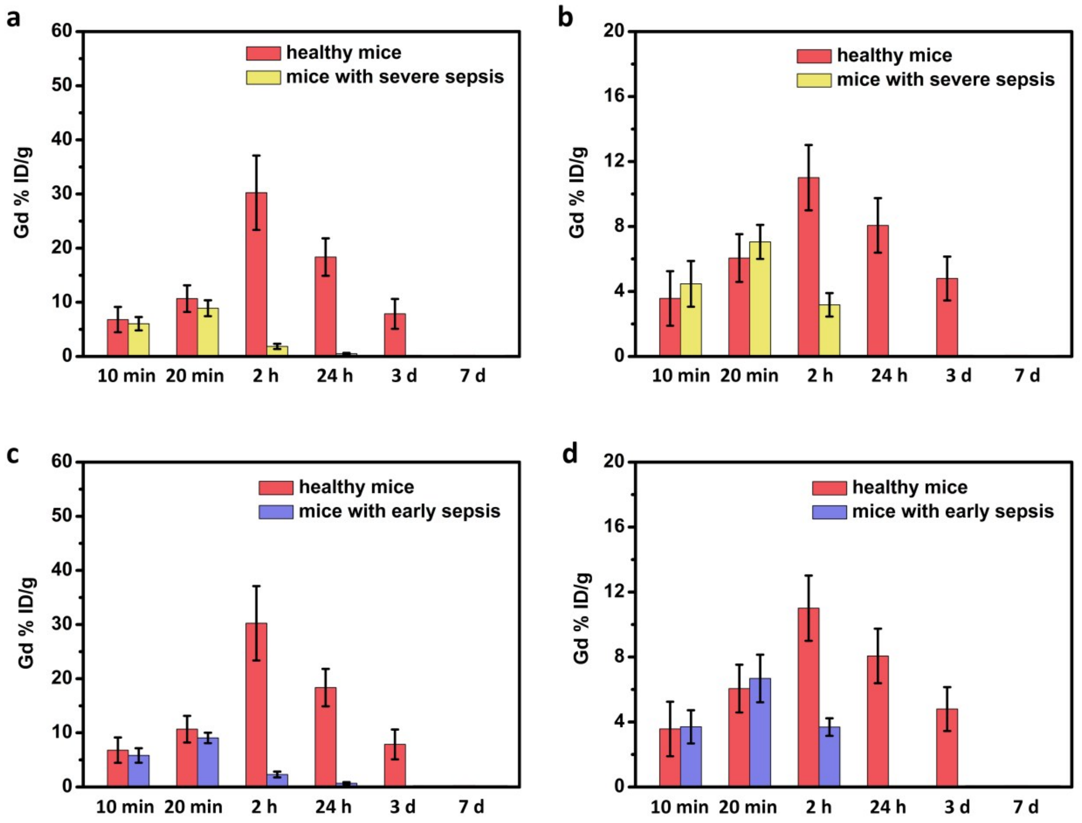

Fig. S12. Time-dependent bio-distributions of Gd in livers of mice with severe sepsis (a) and mice with early sepsis (c). Time-dependent bio-distributions of Gd in kidneys of mice with severe sepsis (b) and mice with early sepsis (d).

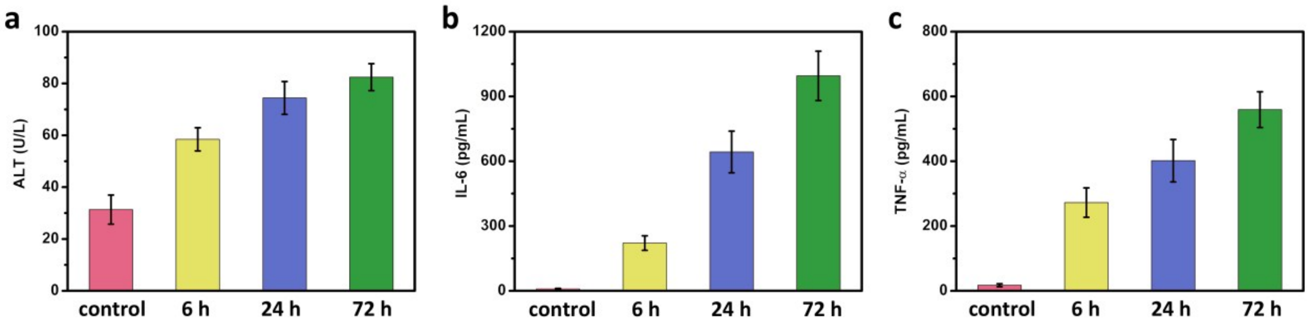

Fig. S13. Time-dependent serum levels of ALT (a), IL-6 (b), and TNF-α (c) of healthy mice post-injection of LPS.

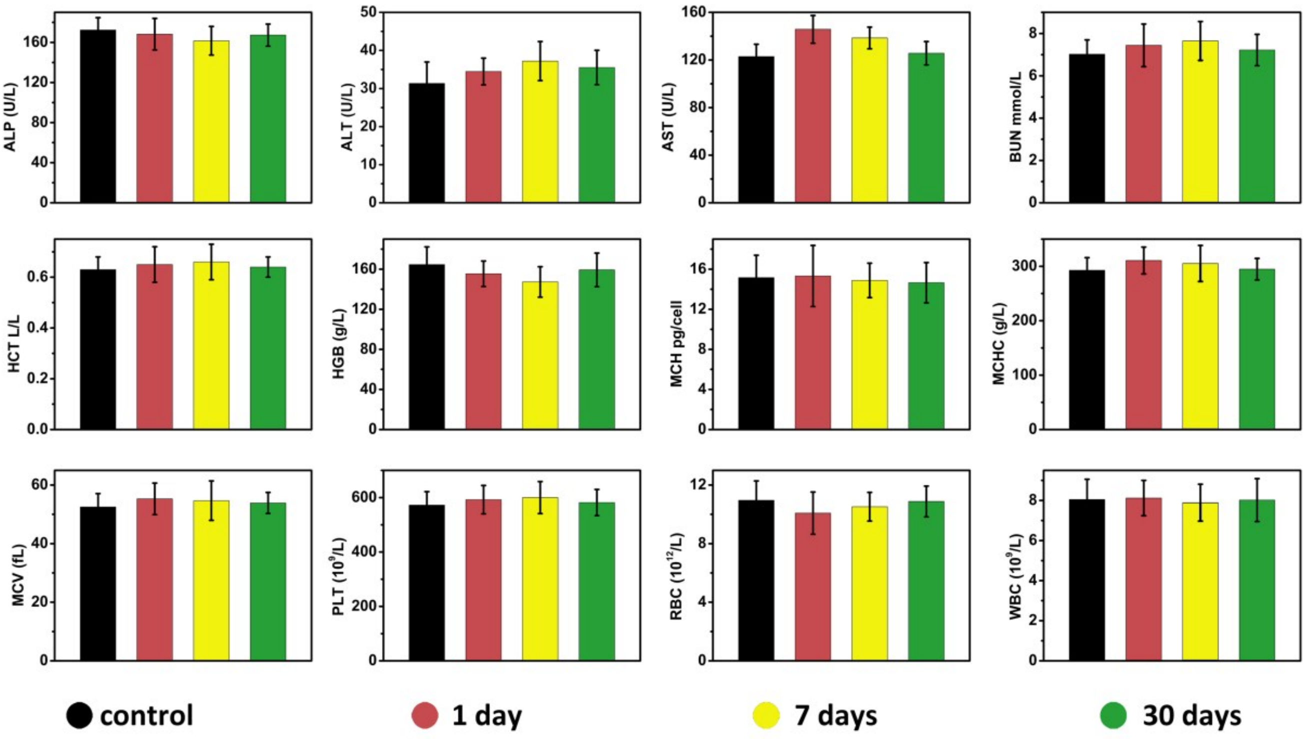

Fig. S14.Blood biochemistry and hematology assays of healthy mice after intravenous administration of ROS CAs. Abbreviation: ALP: alkaline phosphatase; ALT: alanine aminotransferase; AST: aspartate aminotransferase; BUN: blood urea nitrogen; HCT: hematocrit; HGB: hemoglobin; MCH: mean corpuscular hemoglobin; MCHC: mean corpuscular hemoglobin concentration; MCV: mean corpuscular volume; PLT: platelets; RBC: red blood cells; WBC: white blood cells.

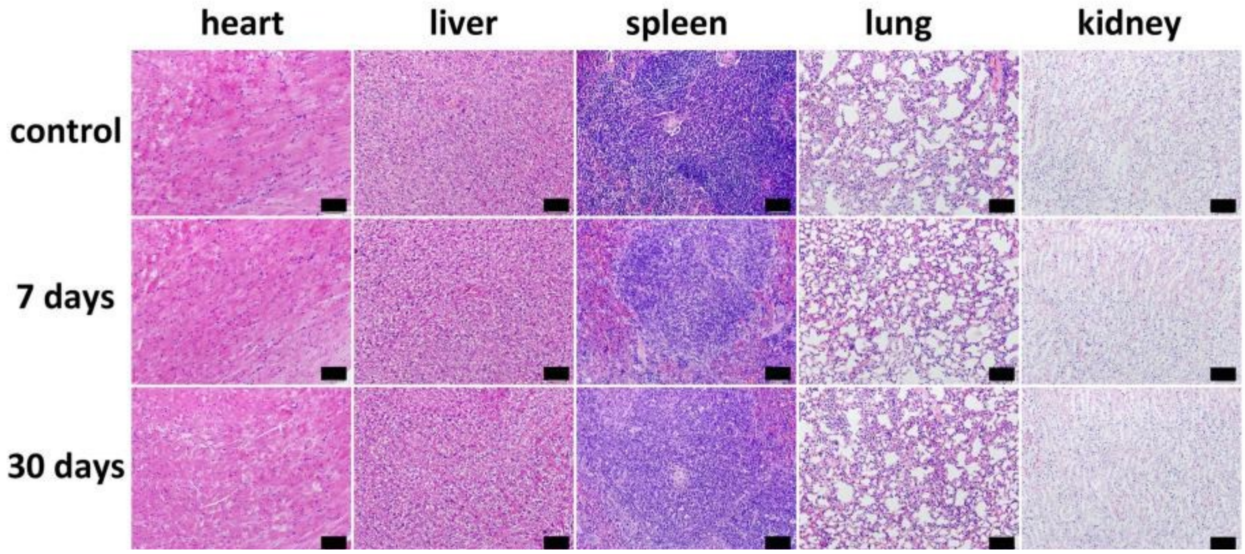

Fig. S15.H&E staining images of major organs in healthy mice after intravenous injection of ROS CAs. Scale bars represented 50 μm.

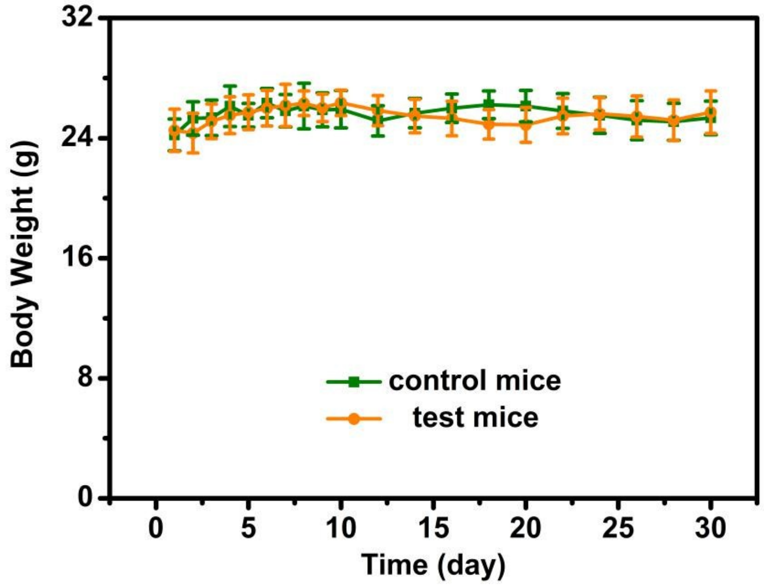

Fig. S16.Body weight changes of healthy mice after intravenous injection ROS CAs.
